# Elite female endurance athletes are at increased risk of atrial fibrillation compared to the general population: a matched cohort study

**DOI:** 10.1136/bjsports-2022-106035

**Published:** 2023-07-10

**Authors:** Nikola Drca, Susanna C Larsson, David Grannas, Mats Jensen-Urstad

**Affiliations:** 1 Department of Medicine Huddinge, Karolinska Institutet, Stockholm, Sweden; 2 Department of Cardiology, Karolinska University Hospital, Stockholm, Sweden; 3 Institute of Environmental Medicine, Karolinska Institutet, Stockholm, Stockholm County, Sweden; 4 Unit of Medical Epidemiology, Department of Surgical Sciences, Uppsala University, Uppsala, Sweden; 5 Division of Biostatistics, Institute of Environmental Medicine, Karolinska Institutet, Stockholm, Sweden

**Keywords:** cardiology, epidemiology, sports medicine

## Abstract

**Objective:**

Previous studies have found that endurance sport activity is associated with an increased risk of atrial fibrillation (AF) in men. However, it remains unclear whether endurance sports also influence the risk of AF in women. We aimed to examine whether participation in endurance sports may affect the risk of AF in female athletes.

**Methods:**

We conducted a retrospective matched cohort study of top Swedish female endurance athletes (n=228) and reference individuals (n=1368) from the general population using the Swedish Total Population Register individually matched with a 6:1 ratio of female athletes. The athlete cohort was created by combining all Swedish women who ran the Stockholm Marathon faster than 3 hours 15 min in any of the races between 1979 and 1991, all women competing in the Swedish athletic national championships in the 10 000 metre race, and the top-ranked Swedish cyclists during the same period. We used the National Patient Register to determine whether the participants were diagnosed with AF.

**Results:**

Mean age at the start of follow-up was 32 (SD±8.5) years. During follow-up (mean 28.8 years; SD±4.4), 33 cases of AF were diagnosed, including 10 (4.4%) among athletes and 23 (1.7%) among references. The HR for female athletes compared with the reference population was 2.56 (95% CI 1.22 to 5.37) in the univariable model and 3.67 (95% CI 1.71 to 7.87) after adjustment for hypertension.

**Conclusion:**

Elite female endurance athletes are at increased risk of AF than the general population.

**WHAT IS ALREADY KNOWN ON THIS TOPIC**

- Endurance sport activity is associated with an increased risk of atrial fibrillation (AF) in men. It is unclear whether endurance sports also influence the risk of AF in women.

**WHAT THIS STUDY ADDS**

- Elite female endurance athletes are at increased risk of AF than the general population.

**HOW THIS STUDY MIGHT AFFECT RESEARCH, PRACTICE OR POLICY**

- Further research is needed to establish the level (duration, frequency and intensity) of endurance training at which AF risk is increased in female athletes.

## Introduction

Physical activity has several positive effects on health. Individuals with a high levels of physical activity have lower blood pressure, lower risk of cardiovascular disease, type 2 diabetes and cancer, better glucose regulation, lipid profiles and mental health, and even increased longevity than less active individuals.^1–3^ Despite these well documented positive effects, studies have shown that physical activity has a J or U-shaped association with the risk of atrial fibrillation (AF).^4–7^ Physical activity at moderate levels is associated with a lower risk of AF, and patients with AF may also benefit from regular physical activity.^8–12^ On the other hand several studies have revealed that vigorous physical activity mainly related to endurance sports is associated with an increased risk of AF in men.^13–17^ This association has not been observed in women and it is unclear whether this is due to a true sex difference or due to a lack of power or adequate exposure to endurance sports training in previous studies involving female athletes.^4 12 18 19^ Different study designs could also affect the ability to differentiate adequate exposure among the participants and could lead to varying study results. Endurance sports have historically been considered inappropriate for women and they have not been allowed to participate in endurance sports events. This could be a reason why older studies were not able to recruit enough women with appropriate exposure to high-intensity physical activity to be able to show an association between physical activity and an increased risk of AF. The first woman to run the Boston Marathon was Roberta Gibb Bingay in 1966 and she had to hide in the bushes near the start until the race began to sneak into the race.^20^ The first women’s marathon race in the Olympic Summer Games was held in Los Angeles in 1984. Long-distance running and other endurance sport activities have become more and more popular among women during the last two decades.^20^ We aimed to examine a cohort of Swedish women with adequate exposure to endurance sports training to see whether a high level of endurance sports training influences the risk of AF in women.

## Methods

### Setting and design

We applied a retrospective matched-cohort study design.

### Patient and public involvement

Patients or the public were not involved in the design, or conduct, or reporting, or dissemination plans of our research.

### Female athlete cohort

**Figure 1:**
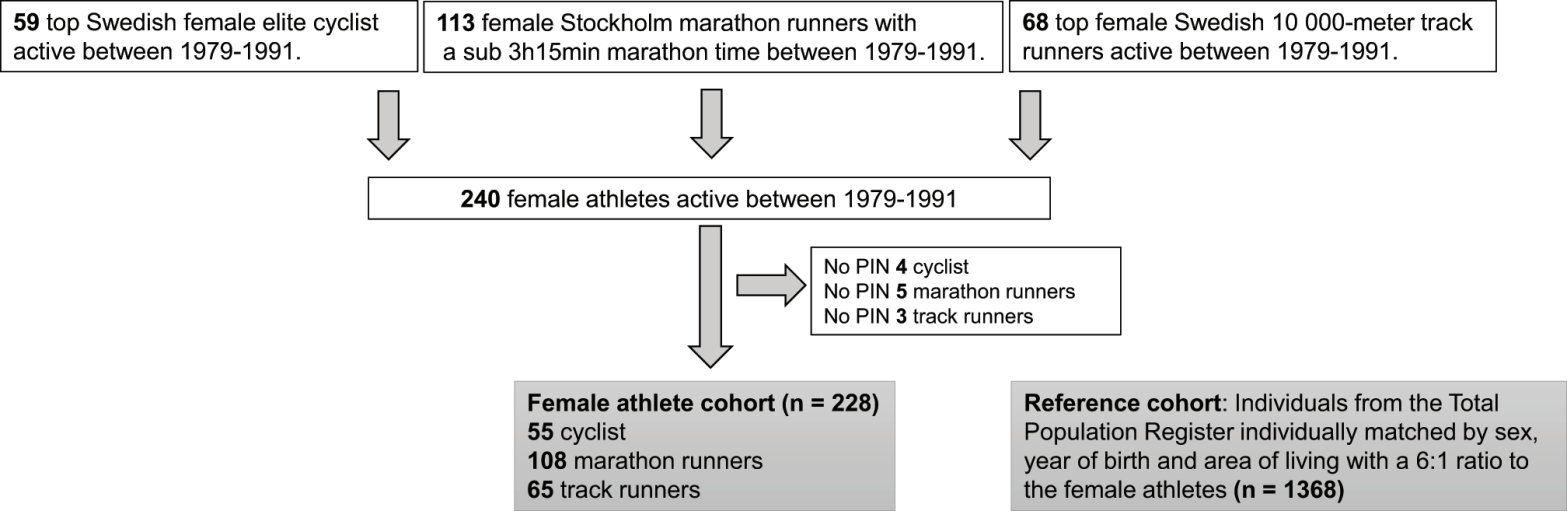
Construction of the female athlete cohort and the reference cohort. PIN, Personal Identiﬁcation Number.

A Swedish female athlete cohort (SFAC) was created by combining all Swedish women who ran the Stockholm Marathon faster than 3 hours and 15 min (3:15 hours) (New York City Marathon qualifying times for women <40 years) in any of the races between 1979 and 1991 with all Swedish women competing in the Swedish athletic national championship in 10 000 metre track running and the top-ranked Swedish road cyclists chosen by the Swedish Cycling Federation during the same period (1979–1991). If a participant was found both among marathon runners and track runners or cyclists she was categorised as a marathon runner. SFAC consisted of 240 women (113 marathon runners, 59 cyclists and 68 track runners). We excluded women with an incorrect or a missing Swedish Personal Identiﬁcation Number (women, n=12 (5 marathon runners, 4 cyclists and 3 track runners)), leaving 228 women for further analysis (figure 1).

### General population comparison cohort

A female reference population comparison cohort was created by individually matching the athlete cohorts with individuals from the Total Population Register (TPR). The TPR was established in 1968 after the data was computerised in 1967. The register contains information on different life events such as birth, death, name change, marital status, family relationships and migration within Sweden as well as to and from other countries.^21^ It has close to full coverage for all births and deaths in Sweden. Emigration data has been estimated to be under-reported up to 0.5% of the Swedish population.

The population was individually matched for sex, age (year of birth) and area of living (municipality) at study entry. For each athlete, six matched controls were collected from the TPR (n=1368) (figure 1).

### Databases

#### Longitudinal integrated database for health insurance and labour market studies

Longitudinal integrated database for health insurance and labour market studies (LISA) is administered by Statistics Sweden and comprises detailed data on health insurance, parental insurance, unemployment insurance, highest attained education, civil status, migration and income at the individual level. LISA covers the adult Swedish population aged ≥16 years since 1990 (since 2010 individuals aged ≥15 years) and it is updated annually.^22^

#### The Swedish National Patient Register

The Swedish National Patient Register (NPR) started as a trial in 1964 and since 1987 has full national coverage of all in-hospital specialist care of somatic and psychiatric care. The NPR contains information about both primary and secondary diagnoses coded according to the Swedish International Classification of Diseases (ICD) system (adapted from the WHO’s ICD classification system). Since 2001, the register also includes outpatient visits from public and private specialist care providers. Since the start of the NPR, primary diagnoses are missing in only 0.8% of somatic care.^23^

#### The Swedish Cause of Death Register

The Swedish Cause of Death Register contains information from 1961. It comprises data on the deaths of all people registered in Sweden. Around 93% of all deaths are reported within 10 days and 100% within 30 days. The cause of death is missing in 1–2% of deaths.^24^

### Case ascertainment

Incident cases of AF were identified through record linkage of the study cohorts to the NPR. The outcome of AF was defined as either a diagnosis of AF or atrial flutter (AFL) because of their close inter-relationship and the difficulties in differentiating between these conditions.

Cases of AF and AFL were identified using the code I48 (I48.0, I48.1, I48.2, I48.3, I48.4, I48.9) from ICD-10, 427D from ICD-9 and 427.92 from ICD-8. The diagnosis of AF in NPR has been validated, in a study by Smith and colleagues, by reviewing ECGs which were available in 98% of the cases. Definitive AF was found in 95% of the cases and no AF in 3%. The 2% with ECGs unavailable had probable AF.^25^

### Covariables

We obtained information on the diagnosis of diabetes, hypertension, ischaemic heart disease (IHD), valvular heart disease, heart failure, chronic obstructive pulmonary disease and hyperthyroidism by linking the study cohorts to the NPR. Educational level was obtained from the LISA register.

### Statistical analysis

The follow-up time for each study participant (athlete and individually matched control) was aggregated from the start of follow-up to the date of diagnosis of AF, death or 31 December 2017 (end of data collection), whichever came first. For female marathon runners, the start of follow-up was from the year they first ran the Stockholm Marathon in less than 3:15 hours. For female track runners, the start of follow-up was from the year they first competed in the Swedish athletic national championship. For female road cyclists, the start of follow-up was from 1991 (since road cyclists did not have any specific competition date that qualified them for inclusion in this study, 1991 was chosen as the start of follow-up). Controls were individually matched (6:1) with athletes and follow-up time started at the same date as the athletes they were matched with. Continuous variables are presented as mean±SD and were compared by using Student’s t-test while categorical variables are presented as frequency (percentage) and compared by the Pearson χ^2^. The Kaplan-Meier method was used to estimate survival curves. HRs with 95% CIs were estimated using Cox proportional hazards models. Comorbidities were included as fixed covariates in the multivariable analyses. Only comorbidities that were diagnosed until 1 year after the diagnosis of AF were included in the analysis. All women were free from AF at the start of follow-up.

Proportional hazards were assessed by Schoenfeld residual plots, however, there were too few AF cases to observe trends over time.^26^

The log-rank test was used to compare survival between groups. In the two multivariable models, we first adjusted for hypertension and then for hypertension, IHD and post-secondary education.

All analyses were conducted using R, V.4.1.2 or higher (R Foundation for Statistical Computing, Vienna, Austria). All statistical tests were two-sided, and p values<0.05 were considered statistically significant.

### Equity, diversity and inclusion statement

This study was designed to include only women because the majority of previous studies were conducted with men as participants. Only women with a correct Swedish Personal Identiﬁcation Number regardless of race/ethnicities or socioeconomic backgrounds were included to enable follow-up through nationwide and population-based registries. The research team included three men (the senior author is a man) and one woman, of whom two are early career researchers.

## Results

**Figure 2:**
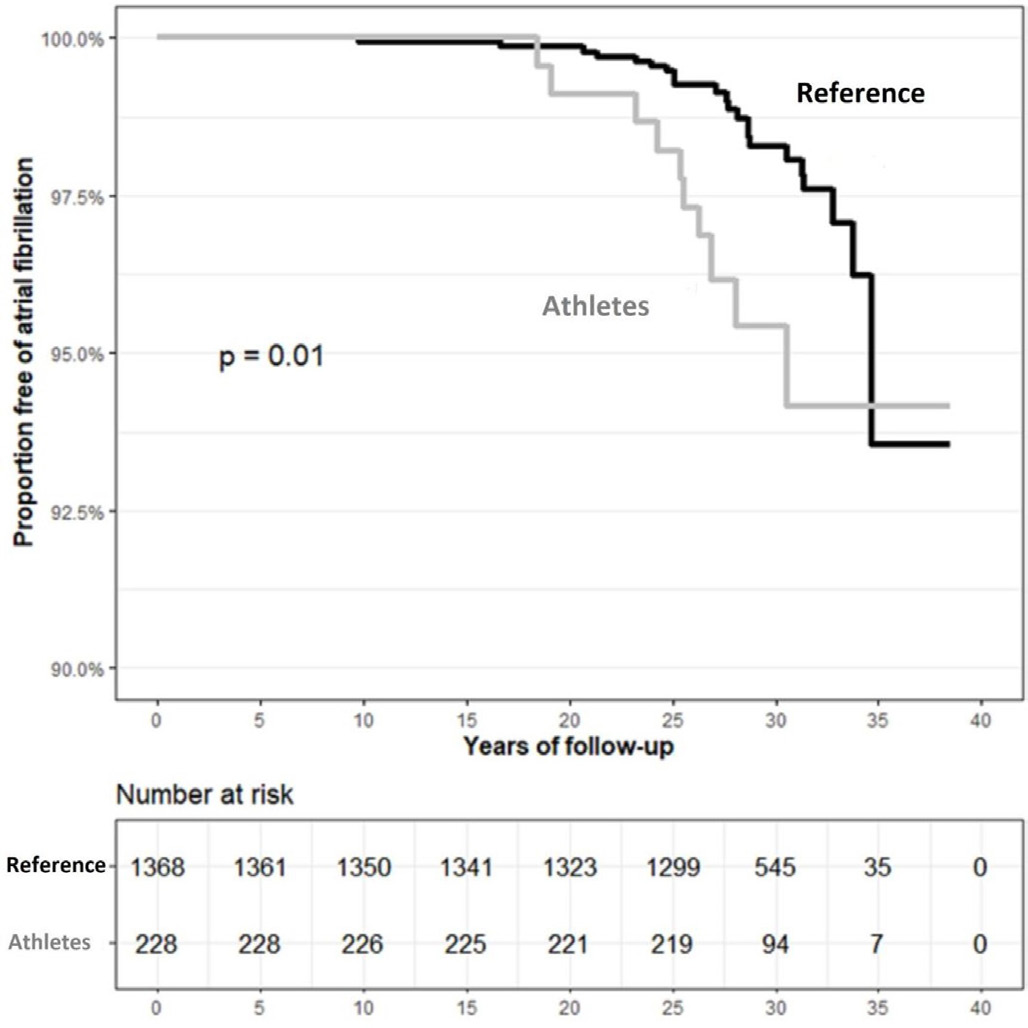
Kaplan-Meier survival curves (unadjusted) (ie, free of atrial fibrillation) for female athletes and the reference population.

The analyses included 228 athletes and 1368 (1:6) reference individuals. The characteristics of the female athletes and reference population cohorts are presented in table 1. Among the participants, the mean age at the start of follow-up was 32 years. There was a significantly higher proportion of post-secondary education among the athletes, and as expected, a significantly lower proportion of hypertension and IHD at the end of follow-up. During follow-up (mean 28.8 years; SD±4.4), 33 cases of AF were diagnosed (7.2 cases of AF per 10 000 person-years), of which 10 (4.4%) cases were diagnosed among athletes and 23 (1.7%) among references. We observed a statistically significant higher risk of developing AF in the athlete cohort compared with the reference population cohort. The HR of AF was 2.56 (95% CI 1.22 to 5.37) in the univariable model (figure 2) and 3.67 (95% CI 1.71 to 7.87) after adjustment for hypertension (table 2). Further adjustment for hypertension, IHD, valvular heart disease and post-secondary education, did not change the results materially but widened the CI (HR 3.63; 95% CI 1.56 to 8.41) (table 2).

**Table 1:** Characteristics of female athletes and matched reference population comparison cohorts

Characteristics	Female athletes* n=228	Reference population n=1368	P value
Age at the start of follow-up, mean (years) (±SD)	32.05 (8.48)	32.05 (8.47)	0.996
Post-secondary education† (%)	46.5	26.8	<0.001
Hyperthyroidism† (%)	0.4	1.7	0.257
Chronic obstructive pulmonary disease† (%)	0.9	1.8	0.493
Diabetes mellitus† (%)	2.2	4.5	0.146
Hypertension† (%)	5.7	14.2	0.001
Valvular heart disease† (%)	1.8	1.0	0.455
Ischaemic heart disease† (%)	0.9	3.8	0.039
Congestive heart failure† (%)	0.9	1.7	0.537

*Marathon runners (n=108), 10 000 metres track runners (n=65) and bicyclists n=55.

†Proportion at the end of follow-up.

**Table 2:** Relative hazards of atrial fibrillation in athletes and reference population

	Age-adjusted HR	Multivariable HR*	Multivariable HR†
Reference population	1.00	1.00	1.00
Female athletes	2.56 (1.22 to 5.37)	3.67 (1.71 to 7.87)	3.63 (1.56 to 8.41)
P value	0.013	0.001	0.001

*Adjusted for age and hypertension.

†Adjusted for age, hypertension, ischaemic heart disease, valvular heart disease and post-secondary education.

## Discussion

This matched cohort study of Swedish female athletes and their matched controls showed that female athletes are at increased risk of AF. The female athletes had an HR of 3.67 to develop AF compared with the controls.

The association between physical activity and AF risk is complex. Previous studies have suggested a U-shaped association, with a decreased risk of AF with low-to-moderate-intensity physical activity and an increased risk with long-term intense physical activity.^8^ Previous studies mainly included men and the few studies involving women have failed to show a statistically significant increased risk with higher intensity physical activity.^12 17–19 27^ It has been hypothesised that this could be due to small sample size and/or lack of proper exposure to long-term intense physical activity in a large proportion of women included in previous studies. In a study of 20 484 adults (50.3% men), Morseth *et al* saw a tendency to a U-shaped pattern in the association between leisure-time physical activity and AF among women but the association was not significant.^5^ Myrstad *et al* studied 89 women with a history of >40 years of regular endurance training and found an increased risk of AF with an OR of 2.18 (95% CI 0.94 to 5.06, p=0.07) compared with women who had never practiced regular exercise. The difference was not statistically significant likely due to a small sample size. The study by Svedberg *et al* on Swedish cross-country skiers competing in Vasaloppet included >82 000 female skiers, where the vast majority participated in the 30 km race (n=66 036) and not the 90 km race, showed a decreased risk of AF among the female cross-country skiers.^18^ Since the association between physical activity and risk of AF is J-shaped or U-shaped, including participant with a more moderate exposure to long-time endurance training could dilute the effect of high-intensity physical activity on AF risk or even show the opposite effect. The present study was designed to ensure proper exposure to long-term physical activity by including only women with documented good results in marathon running, 10 000 metre track-running and cycling. In the entire country of Sweden during a period of 13 years (1979–1991), only 240 Swedish women met the inclusion criteria to be included in the athlete cohort resulting in a rather small sample size with proper exposure. The sample size of our athlete cohort (n=228) was the same as the sample size of the veteran male orienteers in the original study by Karjalainen *et al* from 1998 which was able to show an increased risk of AF among the male orienteers compared with the control group with a relative risk of 5.5 (95% CI 1.3 to 24.4).^13^ In the study by Molina *et al* done on 183 marathon runners, an increased risk of AF was seen compared with their control group with an HR of 8.80 (95% CI 1.26 to 61.29).^15^ These two studies were also of similar design as our study comparing cohorts of athletes (orienteers and marathon runners) to a general population comparison cohort. By investigating female athletes with a proper exposure to high level of endurance sports training we were able to show a significant difference in HR with an HR of 3.67 (95% CI 1.71 to 7.87) for female athletes compared with the general population.

Several different mechanistic factors influencing the development of AF in athletes have been suggested. Increase in parasympathetic tone at rest, increase at sympathetic tone during exercise, increased frequency of premature atrial contractions, development of atrial fibrosis by volume overload and left ventricular hypertrophy are among the most cited mechanisms.^28^ Previous studies have observed a sex difference for atrial and ventricular remodelling and autonomic tone with respect to endurance training in non-elite athletes.^29^ Larger atrial volume and longer signal-averaged P-wave duration, larger left ventricular mass index and greater relative wall thickness and sympathovagal balance as represented by the low/high-frequency power ratio was observed among male athletes. To our knowledge this is the first study that was able to show a statistically significant increased risk of AF among female endurance athletes.

### Research implications

Further research is needed to confirm these results in larger cohorts and establish the level (duration, frequency and intensity) of endurance training at which AF risk increased.

### Limitations

The strength of this study is the inclusion of women who competed in endurance sports at the highest national level, thereby ensuring that women in the athlete cohort had been exposed to a considerable amount of endurance sports training. High-quality nationwide and population-based registries assured complete follow-up. A limitation of this study is the lack of data on actual exposure to endurance sports training since information on training diaries and other reports of training load were unavailable in both the athlete and control group. We used excellent results or high ranking as a proxy for high exposure to endurance sports training. The study is also limited by the small sample size and therefore we were unable to analyse any negative consequences of AF such as stroke, heart failure or death. It also prevented us from analysing the occurrence of different forms of AF (paroxysmal vs persistent). The participants did not have regular medical check-ups and all diagnoses and dates of diagnosis were obtained from the Swedish NPR and comorbidities were not analysed in a time-varying manner in the multivariable model. Some participants could still have AF or some of the comorbidities without it being recorded in the Swedish NPR and this could explain the rather low prevalence specially of comorbidities that usually are asymptomatic such as hypertension. We also cannot entirely exclude the possibility that some participants with asymptomatic AF were misclassified as non-cases and such misclassification should be random and only dilute the association. We further did not include a Swedish male comparison athlete cohort of similar exposure so direct comparison of HRs between men and women is not possible.

In conclusion, this study showed that elite women endurance athletes could have an increased risk of AF than the general female population.

## Data Availability

Data are available upon reasonable request. The data underlying this article cannot be shared publicly due to privacy of individuals that were investigated in the study. The data will be shared on reasonable request to the corresponding author provided that this in accordance with the institutional ethical guidelines as well as regulation and legislation.
